# Coordination of Nanoconjugation with an Antigen/Antibody for Efficient Detection of Gynecological Tumors

**DOI:** 10.1155/2020/6528572

**Published:** 2020-03-30

**Authors:** Xinmei Liu, Xinyuan Yang, Juan Shao, Yufeng Hong, Subash C. B. Gopinath, Yeng Chen, Mang Chek Wey, Yaru Wang

**Affiliations:** ^1^Department of Gynecology, The Fifth Hospital of Xi'an, Xi'an, Shaanxi 710082, China; ^2^Department of Gynecology, The First Affiliated Hospital of Xi'an Jiaotong University, Xi'an, Shaanxi 710061, China; ^3^Department of Gynecology, The Second Affiliated Hospital of Shaanxi University of Traditional Chinese Medicine, Xianyang, Shaanxi 712000, China; ^4^School of Bioprocess Engineering, Universiti Malaysia Perlis, Arau 02600, Perlis, Malaysia; ^5^Institute of Nano Electronic Engineering, Universiti Malaysia Perlis, Kangar 01000, Perlis, Malaysia; ^6^Department of Oral & Craniofacial Sciences, Faculty of Dentistry, University of Malaya, Kuala Lumpur 50603, Malaysia; ^7^Clinical Craniofacial Dental Research Group, Faculty of Dentistry, University of Malaya, Kuala Lumpur 50603, Malaysia; ^8^Department of Gynecology, Xi'an Maternal and Child Health Hospital, Xi'an, Shaanxi 710002, China

## Abstract

Cervical, ovarian, and endometrial cancers are common in the female reproductive system. Cervical cancer starts from the cervix, while ovarian cancer develops when abnormal cells grow in the ovary. Endometrial or uterine cancer starts from the lining of the womb in the endometrium. Approximately 12,000 women are affected every year by cervical cancer in the United States. Squamous cell carcinoma antigen (SCC-Ag) is a well-established biomarker in serum for diagnosing gynecological cancers, and its levels were observed to be elevated in cervical, ovarian, and endometrial cancer patients. Moreover, SCC-Ag was used to identify the tumor size and progression stages. Various biosensing systems have been proposed to identify SCC-Ag; herein, enhanced interdigitated electrode sensing is presented with the use of gold nanoparticles (GNPs) to conjugate an antigen/antibody. It was proved that the limit of detection is 62.5 fM in the case of antibody-GNP, which is 2-fold higher than that by SCC-Ag-GNP. Furthermore, the antibody-GNP-modified surface displays greater current increases with concomitant dose-dependent SCC-Ag levels. High analytical performance was shown by the discrimination against *α*-fetoprotein and CYFRA 21-1 at 1 pM. An enhanced sensing system is established for gynecological tumors, representing an advance from the earlier detection methods.

## 1. Introduction

Gynecological tumors start from the cervix, endometrium, and ovaries of the female reproductive system, and the incidence has recently been increasing among the world population [1–3]. Accurate identification of gynecological tumors is the primary necessity to provide successful treatment for affected patients. Various sensing systems with different techniques have been used to identify the condition and progress of tumors [4–6]. Imaging techniques also support the identification of the advanced stages of gynecological tumors [7]. In addition, serum-based biomarker evaluation is a common strategy to confirm the presence of tumors and their stages [8–10]. Recently, cancer biomarkers have received increasing attention to indicate the level of tumors and the associated issues, even helping follow-up treatment responses [11–13]. It is critical to identify gynecological cancers such as ovarian, cervical, and endometrial cancers at earlier stages. Serum-based biomarkers help to identify the condition of diseases. Serum-based biomarkers originate from the tumor, can appear in the neighboring tissue, and are ultimately excreted into the blood. Moreover, tumor markers have been found to be secreted/released/leaked into the fluids in the interstitium, passing to the lymph and then the bloodstream [11, 13].

Squamous cell carcinoma antigen (SCC-Ag) is a glycoprotein that has been found to be a tumor antigen, showing elevated levels in gynecological tumors. Pretreatment of SCC was found to be related to the disease stage, tumor size, lymph node metastasis, and depth of stromal invasion. Additionally, increased levels of SCC-Ag were shown to have predictive value for tumor diagnosis [6, 7]. The present work detected and quantified the level of SCC-Ag by its antibody for diagnosing gynecological cancer on an interdigitated electrode (IDE) sensing surface assisted by gold nanoparticle conjugation.

The application of nanomaterials in the field of biosensors has improved the detection of biomolecules and reduced the detection limit [14, 15]. Different nanoparticles such as gold, silver, graphene, silica, and copper nanoparticles have been synthesized and used for various kinds of medical applications [16–18]. Among these materials, gold has unique optical properties and is well suited for the biosensor field to improve detection [19]. In general, gold has been used for surface modification/functionalization to immobilize or interact with probes or analyte molecules on sensing surfaces. In some cases, gold is conjugated with a detection probe or analyte molecule to enhance the sensitivity [20–22]. In this research, the conjugation of GNP with SCC-Ag (SCC-Ag-GNP) or anti-SCC-Ag-antibody (SCC-Ag-antibody-GNP) on IDE sensing surfaces was compared.

## 2. Materials and Methods

### 2.1. Reagents and Biomolecules

SCC-Ag was obtained from RANDAX Life Sciences (Malaysia), anti-SCC-Ag antibody was purchased from Next Gene (Malaysia), ethanolamine, (3-aminopropyl)triethoxysilane (APTES), ethanol, PBS (phosphate buffered saline), 16-mercaptoundecanoic acid, and human serum were obtained from Sigma-Aldrich (USA), and N-ethyl-N'-(3-dimethylaminopropyl)carbodiimide hydrochloride (EDC) and N-hydroxysuccinimide (NHS) were procured from GE Healthcare (USA). Gold nanoparticles with a size of 30 nm were purchased from Sigma-Aldrich (USA). *α*-Fetoprotein and CYFRA 21-1 were from MyBioSource (USA). All other reagents used were of analytical grade.

### 2.2. IDE Sensing Surface Preparation

The fabrication of the IDE sensing surface was followed as stated in previous methods using different parameters with chemical and physical surface modifications [23]. Initially, the silicon wafers were oxidized at high temperature, and then, an etching process was carried out with aluminum. The follow-up processes were carried out as described earlier. The surface top layer was coated with zinc oxide. Before starting the surface chemical functionalization, the surface was washed with 1 M KOH (potassium hydroxide at pH 9.0).

### 2.3. Conjugation of Antibody/Antigen on the GNP Surface

To immobilize the SCC-Ag-antibody on the GNP surface, the as-obtained GNPs were linked with 5 mM 16-mercaptoundecanoic acid (16-MDA; contains both -SH and -COOH ends). Then, the samples were centrifuged at high speed to remove the excess 16-MDA. Next, the surface was activated and stabilized by NHS (50 mM) and EDC (200 mM) at a ratio of 1 : 1. Afterwards, 200 nM antibody was added to the activated surface of the GNPs and incubated for 1 h at RT. Then, the unbound antibodies were removed by centrifugation. The GNP-conjugated antibodies (SCC-Ag-antibody-GNP) were washed with PBS to completely remove unbound molecules and stored at 4°C for further use. Similar methods were used for the conjugation of SCC-Ag and GNPs (SCC-Ag-GNP). In this case, different concentrations of SCC-Ag were mixed with GNPs individually with the linker 16-MDA. The unbound SCC-Ag was removed by centrifugation, and SCC-Ag-GNP was used to detect its antibody.

### 2.4. Immobilization of SCC-Ag-Antibody-GNP on the IDE Sensing Surface

To detect SCC-Ag, we compared the antibody on the silane-modified surface of IDE with and without GNP conjugation. Initially, the IDE surface groups were converted to amine groups by dropping 3% APTES diluted in 30% ethanol onto the surface and was kept at room temperature (RT) for 2 h. Next, the amine-modified surface was washed thoroughly with 30% ethanol followed by water. Then, 200 nM antibody with or without GNP was dropped on the amine-modified surface and kept for 30 min at RT to facilitate interactions between the amine surface and antibody/GNP. Finally, the remaining surface sites were blocked by dropping 1 M ethanolamine to avoid any biofouling effects.

### 2.5. Diagnosis of SCC-Ag on SCC-Ag-Antibody Modified Surfaces

SCC-Ag was detected on the SCC-Ag-antibody immobilized surfaces by two methods. Method 1: (i) the surface groups were converted into amine groups by APTES; (ii) 200 nM SCC-Ag-antibody was added; (iii) 1 M ethanolamine was added; and (iv) SCC-Ag-GNP was added.

Method 2: (i) the surface groups were converted into amine groups by APTES; (ii) 200 nM SCC-Ag-antibody-GNP was added; (iii) 1 M ethanolamine was added; and (iv) SCC-Ag was added.

To check the detection limit, lower femtomolar to the lowest picomolar (62.5 fM to 1 pM) levels of SCC-Ag were dropped on the SCC-Ag-antibody-GNP immobilized surfaces from method 1. In the case of method 2, the same concentration of SCC-Ag-GNP was dropped on the SCC-Ag-antibody immobilized surface for comparison. For the specificity analysis, *α*-fetoprotein and CYFRA 21-1 were compared.

### 2.6. Spiking of SCC-Ag into Human Serum and Detection on an Antibody-GNP-Modified Surface

To determine the ability of detecting SCC-Ag in a real biological sample and perform competition experiments, SCC-Ag was spiked into human serum and detected by antibody-GNP conjugates. For that purpose, SCC-Ag with concentrations from 30 to 240 fM was spiked and dropped on an antibody-GNP-conjugated IDE sensing surface. The changes in the current were noted to detect SCC-Ag.

## 3. Results and Discussion

**Figure 1:**
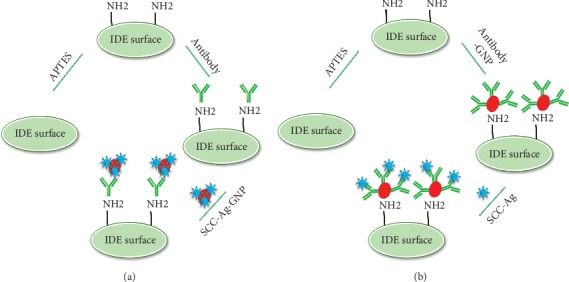
Schematic representation of the detection of SCC-Ag on the amine-modified IDE surface. (a) SCC-Ag-GNP detection on the SCC-Ag antibody-modified surface. The antibody was immobilized on the APTES-modified surface, and then, SCC-Ag-GNP interacted with the antibody. (b) SCC-Ag-antibody-GNP was immobilized on the APTES-modified surface, and then, SCC-Ag interacted on the surface, enabling its detection.

Identifying gynecological tumors, such as ovarian, cervical, and endometrial cancers, is mandatory to treat these diseases and avoid spreading to other organs of the body. Serum-based biomarkers help to identify tumors effectively and are considered to have diagnostic potential. SCC-Ag is one of the proven biomarkers in serum for tumors, and its concentration is elevated in most gynecological tumors [24]. In this work, SCC-Ag was detected by assistance with its antibody on an amine-modified IDE sensing surface. IDE sensors have been shown to efficiently detect various diseases with highly specific interactions of biomolecules [25, 26]. To improve the detection limit, a gold nanoparticle- (GNP-) conjugated antibody or GNP-conjugated SCC-Ag was used, and these two methods were compared on the IDE sensing surfaces. GNPs have been used in different ways to improve diagnostic systems, mainly by surface modification and conjugation with analytes or target molecules. Surface modification helps to evenly arrange molecules on the sensing surface and helps increase the number of biomolecules immobilized on the surface of the sensor. Since it has been proven that the proper arrangement of biomolecules on the surface of the sensor improves the detection system [27, 28], we also expected the proper arrangement of biomolecules with the help of GNPs to improve the detection of SCC-Ag. In the other case, when the detection molecule is conjugated with GNPs, a higher number of biomolecules bind to the GNP surfaces, so in this work, the conjugation of SCC-Ag to GNPs improves the binding of its antibody on the IDE surface. The above two methods were compared with similar concentrations of SCC-Ag.  Figure 1 shows a schematic representation of the detection strategy for SCC-Ag on amine-modified surfaces. In the case of method 1, SCC-Ag-antibody was immobilized on the amine-IDE surface, and then, SCC-Ag-GNP was used to detect the level of SCC-Ag (Figure 1(a)).  Figure 1(b) explains method 2 for the detection of SCC-Ag on the SCC-Ag-antibody-GNP-immobilized surface. The SCC-Ag-antibody-GNP was bound on the sensing surface by amine interactions, and then, SCC-Ag could interact; this happens because the GNPs, which have a negative surface charge, bind on the positively charged amine-modified surfaces by electrostatic interactions [29].

### 3.1. Comparison of Antibody Immobilization on the IDE Surface with and without GNPs

**Figure 2:**
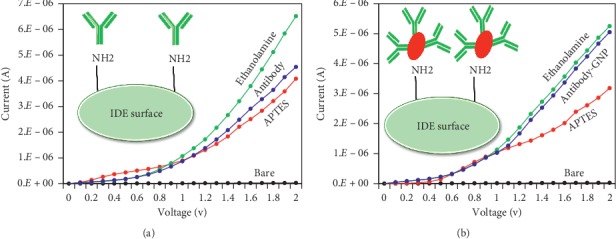
Comparison of the antibody immobilization process on APTES-modified IDE sensing surfaces: (a) without GNP; (b) with GNP. Antibody-GNP-modified surfaces show greater changes in current increases than those without GNPs.

As explained above, two different kinds of probe modifications were prepared on the IDE surface by SCC-Ag-antibody: with and without GNPs. As shown in Figure 2(a), with the bare surface, the maximum current level was 2.66*E*^−06^; when APTES was added on the surface, the current was increased to 4.08*E*^−06^. This result confirms the amine modification on the IDE surface. Next, when 200 nM antibody was dropped on the surface, the current was increased to 5.86*E*^−06^ and gave a difference of 1.78*E*^−06^, and then, 1 M ethanolamine, as a blocking agent, caused the current to increase to 6.52*E*^−06^. These results clearly show the proper binding of the probe SCC-Ag-antibody on the IDE surface. In the case of method 2, after APTES treatment, 200 nM antibody-GNP was added, and the current increased from 3.18*E*^−06^ to 5.05*E*^−06^ (Figure 2(b)). Thus, the current difference was 1.87*E*^−06^, which is an almost 1.6-fold increase compared with the current change when conjugating the antibody with GNPs. This increase is due to the larger number of antibodies immobilized on the surface of GNPs, which leads to more immobilized antibodies on the IDE surface for the sensing interactions.

### 3.2. Detection by Conjugation of SCC-Ag-Antibody-GNP/SCC-Ag-GNP: Comparison

**Figure 3:**
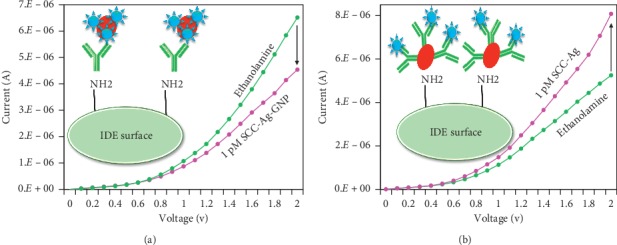
Detection of 1 pM SCC-Ag by the methods: (a) SCC-Ag-GNP with the antibody; (b) SCC-Ag on the SCC-Ag-antibody-GNP surface.

It was proved that SCC-Ag-antibody-GNP shows higher current changes than SCC-Ag-antibody does. Then, the detection of SCC-Ag on SCC-Ag-antibody-modified IDE sensing surfaces (method 1) and SCC-Ag-antibody-GNP-modified IDE sensing surfaces (method 2) was performed.  Figure 3(a) shows 1 pM SCC-Ag-GNP detection on the antibody-immobilized surface, clearly showing a current change from 6.52*E*−06 to 4.54*E*−06 with a difference of 1.98*E*^−06^. In the case of method 2, the similar concentration of 1 pM SCC-Ag on the SCC-Ag-antibody-GNP-immobilized surface shows a current increase from 5.25*E*−06 to 8.08*E*−06, and the difference was found to be 2.83*E*−06 (Figure 3(b)). From this result, it was concluded that method 2 (SCC-Ag on SCC-Ag-antibody-GNP) shows greater changes than method 1 (SCC-Ag-GNP on SCC-Ag-antibody). This result might be due to the larger number of probes available with SCC-Ag-antibody-GNP bound on the APTES-modified IDE surface, leading to a larger amount of SCC-Ag binding.

### 3.3. Limit of SCC-Ag Detection

**Figure 4:**
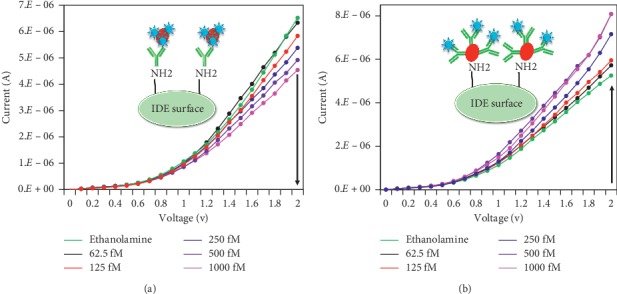
Limit of SCC-Ag detection. (a) SCC-Ag-GNP at concentrations from 62.5 fM to 1 pM was detected with the antibody. (b) SCC-Ag at concentrations from 62.5 fM to 1 pM was detected on antibody-GNP surfaces. SCC-Ag-antibody-GNP-modified surfaces show a greater response of current at all concentrations of SCC-Ag tested.

The limit of SCC-Ag detection was also determined with both methods 1 and 2. For this assessment, SCC-Ag was titrated from 62.5 fM to 1 pM and detected by both methods 1 and 2. In the case of method 1, with a 62.5 fM concentration, the current showed a change from 6.52*E*−06 to 6.33*E*−06. With increasing concentrations of SCC-Ag, the concomitant current levels gradually decreased. At 125 fM SCC-Ag, the current was 6.06*E*−06; at 250 fM, it was 5.38*E*−06; at 500 fM, it was 4.92*E*−06; and at 1 pM, it was 4.54*E*−06. (Figure 4(a)). In the case of method 2, at 62.5 fM SCC-Ag, the current increased from 5.25*E*−06 to 5.72*E*−06; at 125 fM, it was 5.95*E*−06; at 250 fM, it was 7.16*E*−06; and at 500 fM and 1 pM, a saturation current of 8.08*E*−06 was observed (Figure 4(b)). Comparing methods 1 and 2, method 2 shows greater changes in the current increase at all the concentrations of SCC-Ag tested.

**Figure 5:**
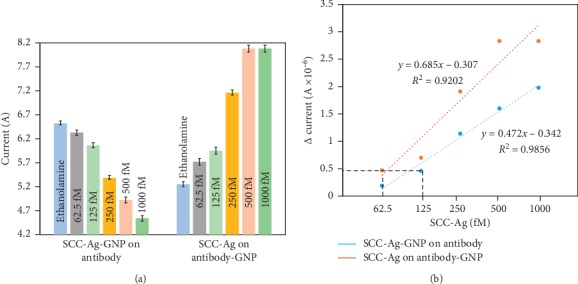
(a) Comparison of the difference in current changes with various concentrations of SCC-Ag. Methods of SCC-Ag-GNP on the antibody and SCC-Ag on SCC-Ag-antibody-GNP surfaces were considered. Both methods show significant changes in the current from 62.5 fM and saturated at 1 pM SCC-Ag. (b) Linear regression analysis for the interaction of different concentrations of SCC-Ag-GNP with ASC-Ag-antibody and SCC-Ag with SCC-Ag-antibody-GNP. The sensitivity was calculated based on 3*σ* as indicated. The calculated sensitivities are 125 and 62.5 fM for methods 1 and 2, respectively.

Figure 5(a) shows the comparison of the difference in current changes with all the concentrations of SCC-Ag by methods 1 and 2. It was noticed that compared with method 1, method 2 showed gradual increases in the current changes at all the tested concentrations of SCC-Ag. This is in another result from method 1, which might be due to the proper arrangement of SCC-Ag-antibody-GNP on the IDE sensing surface. In method 2, the apparent current change was noticed from 60 fM, while in method 1, it was noticed from 120 fM.  Figure 5(b) presents a linear regression analysis of the interaction of different concentrations of SCC-Ag with its antibody; the sensitivity was calculated based on 3*σ*. It was clear by these analyses that the sensitivity attained by methods 1 and 2 was 120 and 62.5 fM, respectively. These ranges are comparable, showing better performance than that of the currently available sensors (Table 1).

### 3.4. Detection of SCC-Ag-Spiked Human Serum on the Antibody-GNP-Modified IDE Sensing Surface

**Figure 6:**
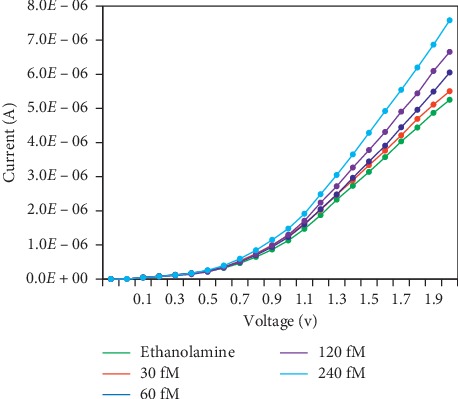
Spiking of SCC-Ag into human serum. SCC-Ag concentrations from 30 to 250 fM were spiked in human serum and detected by SCC-Ag-GNP. Apparent changes were noted in comparison with the condition of spiking into PBS.

After confirming the SCC-Ag detection limit, to evaluate the ability of SCC-Ag detection in the biological sample, different concentrations of SCC-Ag were spiked into human serum and detected by antibody-GNP conjugates. As shown in Figure 6, when 30 fM SCC-Ag was spiked in serum, the current did not significantly change, but the change was better than that of the SCC-Ag-spiked PBS sample. When the concentration was increased to 60 fM, the current clearly increased. Furthermore, with increasing concentrations of SCC-Ag, the current levels also gradually increased. As a well-known fact, serum has large quantities of proteins and biomarkers. Albumin and globulin are the predominant proteins in the serum, at 45 mg·mL^−1^ and 20–35 mg·mL^−1^, respectively. In addition, the commonly recognized IgM level is 0.75–3.0 mg·mL^−1^, and the IgG level is 6.5–18.50 mg·mL^−1^. Considering these higher levels of interferents/competitors, the above assay is competition-based. It has been reported that an SCC-Ag level of 2 ng/mL is the upper limit of normal individuals, and the current method offering lower to higher levels of detection of SCC-Ag helps to distinguish between normal and cancer patients.

## 4. Conclusion

Gynecological tumors in the female reproductive system mainly occur in the form of cervical, ovarian, and endometrial cancers. They cause various health issues, and the later stage of these tumors spread to other parts of the body, making it mandatory to identify the tumor at earlier stages. Early diagnosis will help improve treatment and avoid metastasis. Squamous cell carcinoma antigen (SCC-Ag) is a serum-based biomarker that has been found at elevated levels in gynecological tumors. In this work, SCC-Ag was detected on an amine-modified interdigitated electrode sensor assisted by the antibody. Gold nanoparticle-conjugated biomolecules were used to improve the detection. Two methods, namely, SCC-Ag-GNP on SCC-Ag-antibody (method 1) and SCC-Ag on SCC-Ag-antibody-GNP (method 2), were compared for detection. It was found that method 2 shows better sensitivity with a higher increase in current changes at all concentrations of SCC-Ag tested and worked well in the SCC-Ag-spiked serum samples. Such methods with gold-conjugated probes/targets will help to identify and quantify the severity level of gynecological tumors.

## Figures and Tables

**Table 1:** Comparison of methods for the detection of SCC-Ag.

Detection method	Limit of detection	Reference
Electrochemical sensor	10 pM	[24]
Electrochemiluminescent sensor	0.4 pg/mL	[30]
Surface plasmon resonance	0.1 pM	[31]
Surface-enhanced Raman scattering	7.16 pg/mL	[32]
Electrochemical sensor	80 pM	[33]
Field effect transistor	10 fg/mL	[34]
Interdigitated electrode sensor	10 fM	Current study

## Data Availability

All the data and materials are available without restriction.
